# Immersive 3D Virtual Reality–Based Clip Sizing for Thoracoscopic Left Atrial Appendage Closure

**DOI:** 10.1177/15569845221114344

**Published:** 2022-08-01

**Authors:** Frank van Schaagen, Yvar P. van Steenis, Amir H. Sadeghi, Ad J.J.C. Bogers, Yannick J.H.J. Taverne

**Affiliations:** 1 Department of Cardiothoracic Surgery, Thoraxcenter, 6993 Erasmus University Medical Center, Rotterdam, The Netherlands

**Keywords:** atrial fibrillation, stroke prevention, left atrial appendage closure, virtual reality

## Abstract

**Objective:** Surgical left atrial appendage (LAA) closure using epicardial clips has become popular in stroke prevention in patients with atrial fibrillation. Optimal placement of the clip is essential to achieve complete LAA occlusion and to prevent complications due to compression of the circumflex artery. We determine the added value of immersive virtual reality (VR) in accurately assessing LAA base size and selection of an appropriately sized clip. **Methods:** We studied the feasibility of measuring the LAA base using VR and conventional computed tomography (CT). A retrospective analysis was performed of LAA base measurements in 15 patients who had undergone thoracoscopic LAA clipping. Subsequently, we compared the placed clip size with imaging-acquired LAA base size to retrospectively evaluate intraprocedural sizing. **Results:** We successfully applied a VR platform to measure LAA base size. The median LAA base size measured in CT (23.8 mm, interquartile range [IQR] 22.3 to 26.4 mm) and intracardial virtual reality (23.4 mm, IQR 21.6 to 25.5 mm) did not differ significantly (*P* = 0.416). VR measurements of the LAA base in surgeon's view (25.7 mm, IQR 24.2 to 29.2) were significantly higher than those of 2-dimensional CT (*P* = 0.037) and intracardial 3-dimensional (3D) VR (*P* < 0.05) measurements. All measurements differed significantly with placed clip sizes (*P* < 0.05). There were no clip malpositioning-related events. **Conclusions:** Immersive VR is a feasible method for obtaining anatomical information on LAA base size. In this retrospective analysis, CT and VR-based measurements of LAA base size differed significantly from intraoperatively placed LAA clips, indicating potential oversizing when measured intraoperatively. Using intuitive 3D VR-based imaging might be a useful method to assist in accurate preprocedural sizing of LAA base and can potentially prevent oversizing.

**Central Message**

Immersive virtual reality is a feasible method to obtain anatomical information on LAA base size. Using intuitive 3D virtual reality-based imaging might be a useful method to assist in accurate preprocedural sizing of the LAA base and can potentially prevent oversizing.

## Introduction

Over the past few years, left atrial appendage (LAA) exclusion devices, such as the AtriClip (AtriCure, Mason, OH, USA), have emerged and been shown to be of added value to oral anticoagulation for stroke prevention in patients with atrial fibrillation (AF).^1,2^ In addition, LAA exclusion might be beneficial for stroke prevention in patients with a contraindication or intolerance for oral anticoagulation who are undergoing cardiac surgery.^3^ Several studies have demonstrated the AtriClip to be a safe and effective LAA closure tool.^1–5^ Although low rates of device-related complications have been described in the literature, malpositioning of the AtriClip can result in complications.^6^ For instance, oversizing could compress or induce damage to the surrounding blood vessels, such as the left main coronary artery and pulmonary artery, whereas undersizing could compromise the left circumflex artery by folding the tissue or result in incomplete closure of the LAA.^6^ Thus, accurate and precise measurements of the LAA and subsequent proper positioning of the clip are essential.

The use of virtual reality (VR) and 3-dimensional (3D) imaging tools have recently gained attention in the field of surgical training, planning, and intraoperative navigation.^7,8^ Within the field of cardiothoracic surgery, VR has been evaluated in different settings for planning and simulation of surgical procedures.^7,9^ At this stage, it is possible to convert 2-dimensional (2D) computed tomography (CT) scans into a 3D model that can be viewed in a 3D immersive VR environment. This allows the user to manipulate and interact with the 3D CT model of a patient. VR-based 3D preoperative planning might improve the accuracy of AtriClip sizing for LAA closure and reduce the risk of complications. To study the feasibility of 3D VR preoperative planning, we conducted a retrospective analysis with a cohort of 15 patients who had undergone LAA closure during thoracoscopic ablation procedures in our center. The current study aim was to determine the feasibility of using 3D VR software to measure LAA base size for adequate AtriClip selection. In addition, we retrospectively analyzed the applicability of VR in providing measurements when compared with conventional sizing (using a ruler and the manufacturer's selection guide) and the previously applied AtriClips.

## Methods

### Inclusion Criteria and Patients

Inclusion criteria were (1) patients 18 years of age or older, (2) thoracoscopic LAA closure successfully performed at our institution, and (3) at least 1 CT scan available to the researchers. Between August 2018 and August 2020, 15 patients met inclusion criteria and were included in the study. Written informed consent was obtained. This study was approved by the Medical Ethical Committee of Erasmus University Medical Center, Rotterdam, The Netherlands (MEC-2020-0787, October 23, 2020).

### Surgical Approach

In all patients, closure of the LAA was part of a thoracoscopic ablation procedure for symptomatic, drug-refractory AF, as previously described by Van Laar et al.,^10^ without a trigonum line. Right-sided and left-sided thoracoscopy was used for right-sided and left-sided pulmonary vein isolation (PVI) by means of a bipolar clamp (AtriCure). A box lesion was created by connecting the PVI lines with the bipolar unidirectional Coolrail device (AtriCure). The left-sided PVI was connected to the LAA. The base of the LAA was measured, and the correct size AtriClip was chosen using the Gillinov–Cosgrove Selection Guide (AtriCure). After clip placement, transesophageal echocardiography was used to confirm complete closure of the LAA, and electrocardiography combined with echocardiography was used to exclude signs of regional wall ischemia possibly induced by circumflex artery occlusion.

### 3D Environment

**Fig. 1.**
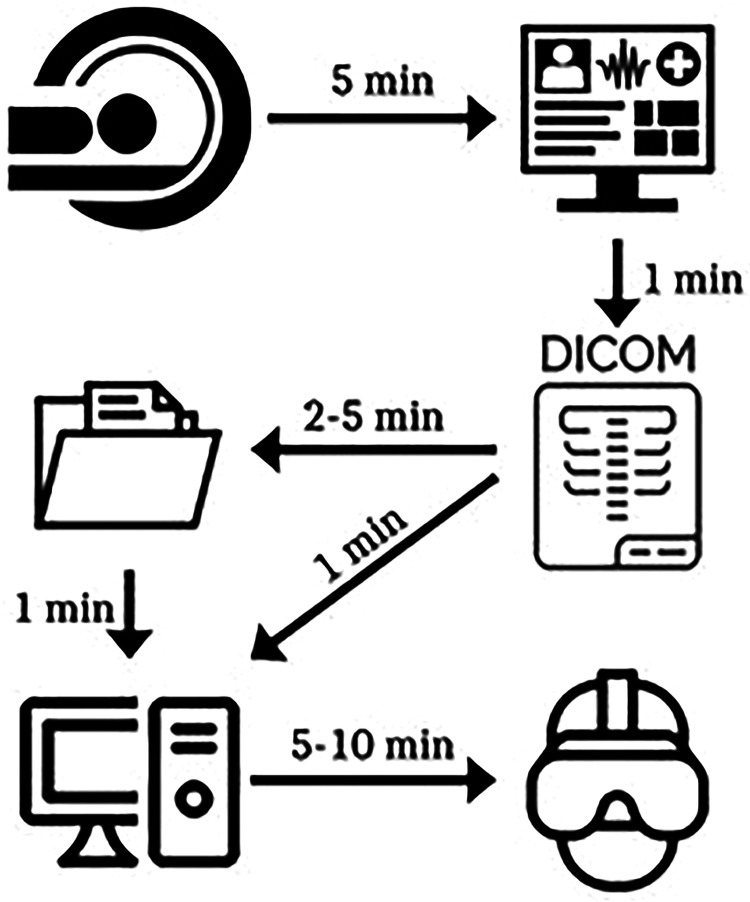
Schematic representation of the creation of a 3-dimensional environment. (a) A preoperative conventional contrast CT scan, (b) available from the electronic health record, (c) was exported as a DICOM file. (d) A segmentation file was made, and (e) together with the DICOM file, they were imported into MedicalVR. (f) This allowed virtual reality analysis of the CT scan. Time indications are given in minutes. The total time was about 15 to 20 minutes per CT scan. CT, computed tomography; DICOM, Digital Imaging and Communications in Medicine.

Conventional contrast-enhanced CT scans were used as a basis for the 3D reconstructions. The CT scans were exported from the electronic patient records as a Digital Imaging and Communications in Medicine (DICOM) file. This file was used to create a segmentation of the left atrium (according to a previously published protocol), ensuring the entire LAA was clearly visible.^7^ In addition, a segmentation of the LAA wall was made. Both the DICOM file and the segmentation file were imported into our MedicalVR workstation (Amsterdam, The Netherlands). As such, 3D reconstruction and immersive evaluation of the 2D CT scan was possible. Figure 1 schematically shows the process of creating the 3D environment.

### Data Collection

Data on the size of the AtriClip and postoperative outcomes were retrospectively collected. LAA size, measured at the ostium of the LAA, in both 2D conventional CT and 3D VR were retrospectively measured using preoperative CT scans. In 2D CT, the ostium was measured in the axial view from the base of the Coumadin ridge to the proximal aspect of the left circumflex artery, which has been described by Xu et al. for Watchman device placement.^11^

**Fig. 2.**
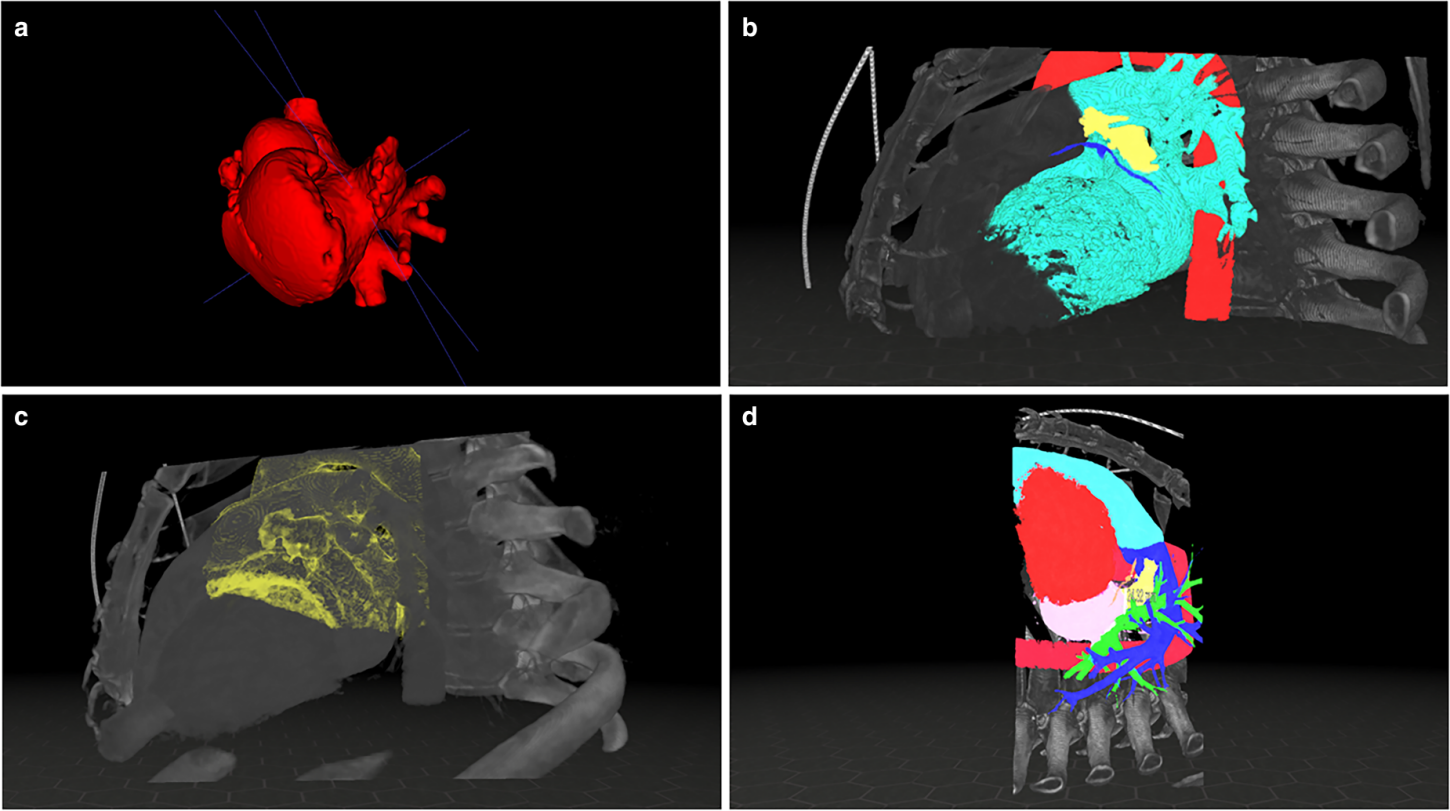
Screen captures of (a) segmentation, (b) overview of the thorax region with colors indicating aorta (red), coronary arteries (dark blue), LAA (yellow), and segmentation (light blue), (c) surgeon's view of LAA using wall segmentation (yellow), and (d) measurement of LAA (yellow). LAA, left atrial appendage.

In 3D VR, the user viewed the left atrium from inside the heart, such that a clear view of the pulmonary veins, mitral valve, and LAA ostium was achieved. Using a virtual measuring tool, 3 measurements of the longest axis of the ostium were made, parallel to the location of epicardial clip placement. In this cohort, measurements ranged from 17.17 to 32.47 mm. The average of these 3 measurements was used in further analysis. Finally, a second measurement in 3D VR was made from the surgeon's virtual view. A segmentation of the epicardial wall of the LAA was loaded into VR, and the base of the LAA was measured along the position where the AtriClip would be placed surgically. This measurement was also performed 3 times, and the average was used in further analysis. Measurements ranged from 19.82 to 37.23 mm. Figure 2 depicts intra-VR screen captures of 3D CT reconstructions, showing the orientation and measurement tool.

### Statistics

Continuous variables are given as median and interquartile range (IQR). Categorical variables are given as frequencies and percentages. A statistical analysis was performed of the accuracy of LAA measurement, comparing 3D VR (both intracardial and surgeon's view measurements) with 2D CT and the actual size of the placed AtriClip with 2D CT and 3D VR. A paired *t*-test of LAA size measurements was performed. A *P* value of less than 0.05 was considered significant. All statistical analyses were performed in Microsoft Excel (Microsoft, Redmond, WA, USA).

## Results

### Patient Population

Fifteen patients who met inclusion criteria were retrospectively identified. All operations were performed between July 2018 and July 2020. All patients had previously undergone a catheter ablation for PVI. Nine of the included patients were male (60%), and 6 patients were female (40%). The median age was 61 years (IQR 59.5 to 67.0 years). Baseline characteristics and a detailed outline of the indications for LAA exclusion and thoracoscopic ablation are presented in Table 1.

**Table 1.** Baseline Characteristics and Indications for LAA Exclusion Surgery.

Patient characteristics	Total sample (*N* = 15)
Male sex	9 (60)
Age, years	61 (59.5–67)
Indication for surgery	
Paroxysmal AF	2 (13)
Persistent AF	12 (80)
Long-standing persistent AF	1 (7)

Abbreviations: AF, atrial fibrillation; LAA, left atrial appendage.

Data are presented as *n* (%) or median (IQR).

### Adverse Events

All patients underwent successful LAA closure. Perioperatively, no adverse events related to misplacement (e.g., bleeding, myocardial infarction), oversizing, or undersizing of the AtriClip occurred. None of the patients experienced a cerebrovascular accident perioperatively.

### AtriClip Size and LAA Base Measurements

**Fig. 3.**
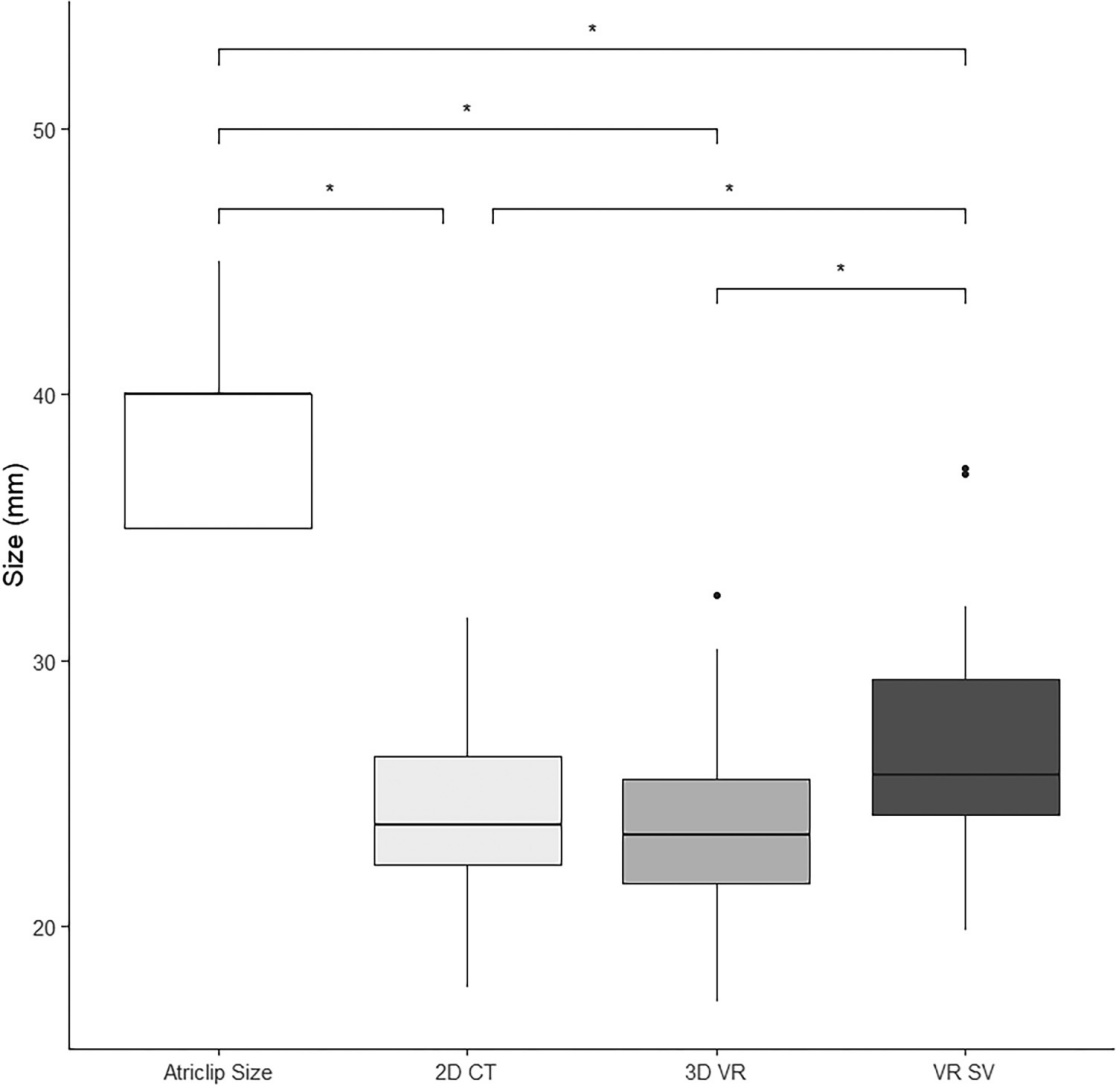
Boxplots of placed AtriClip size, 2D CT, 3D VR intracardial view, and surgeon's view virtual reality measurements. Center lines show the medians, box limits indicate the 25th and 75th percentiles, whiskers extend 1.5 times the interquartile range from the 25th and 75th percentiles, outliers are represented by dots, and significance is indicated by their respective bars (*N* = 15 sample points). 2D CT, 2-dimensional computed tomography; 3D VR, 3-dimensional virtual reality intracardial view; VR SV, virtual reality surgeon's view.

In the analyzed patient population, 3 different AtriClip sizes were used to close the LAA. These were either a 35 mm (6 patients, 40.0%), 40 mm (7 patients, 46.7%), or 45 mm (2 patients, 13.3%) AtriClip. The median LAA base measurement in conventional 2D CT was 23.8 mm (IQR 22.3 to 26.4 mm). In 3D VR, the median of all measurements was 23.4 mm (IQR 21.6 to 25.5 mm) using the intracardial view and 25.7 mm (IQR 24.2 to 29.2 mm) using surgeon's view. In Figure 3, a boxplot of all measurements and AtriClip size is shown. AtriClip sizes were significantly different from all preoperative measurements (2D CT, intracardial 3D VR, and surgeon's view 3D VR; all *P* < 0.05). There was no significant difference between 2D CT and intracardial 3D VR (*P* = 0.416). Surgeon's view 3D VR showed significantly higher measurements of LAA base size compared with both 2D CT (*P* = 0.037) and 3D VR (*P* < 0.05).

## Discussion

In this study, we evaluated the feasibility and applicability of 3D VR to assist in LAA base sizing for concomitant LAA closure in the context of thoracoscopic AF ablation. We have shown that intracardial 3D VR measurements did not differ significantly from 2D CT measurements, and both differed significantly from AtriClip size. Surgeon's view 3D VR, arguably the better approximation of AtriClip size than intracardial 3D VR, was significantly different than 2D CT and intracardial 3D VR but also from AtriClip size. Finally, our results indicate that the technique used to determine LAA ostium size in 2D CT, as described by Xu et al.,^11^ is potentially an adequate method to determine LAA ostium size, given that we reported similar values when intracardial 3D VR and 2D CT measurements were compared (*P* = 0.416).

Interestingly, both 2D CT and intracardial 3D VR measurements showed significantly lower LAA sizes when compared with the actual placed AtriClip size. We can identify 2 main reasons for this discrepancy. First, the measurements performed in this study were of the internal lumen of the LAA ostium, both in 2D CT and in intracardial 3D VR. The AtriClip is placed epicardially and is thus a few millimeters larger than the internal lumen. Second, the AtriClip is placed slightly away from the ostium. This might result in a larger AtriClip size compared with ostium size. Both obstacles could be addressed using the surgeon's view 3D VR; segmentation of the LAA wall ensures measurement of the correct structure, and by using this view, a more accurate LAA base size can be measured along the exact angle that the AtriClip would have been placed. Although surgeon's view 3D VR measurements were significantly higher than 2D CT and intracardial 3D VR measurements, they were still significantly lower than placed AtriClip sizes. Consequently, we hypothesize that oversized clips have been used in almost all cases of LAA closure, due to less accurate intraoperative measurement with the AtriClip sizing tool. Possible explanations for these inaccurate measurements, in reality, are that a beating heart, limited space for measuring, and a view of the entire LAA base that is not ideal can all complicate the accurate measurement of the LAA base. A recent case report by Hassan et al.^6^ highlighted the difference between intraoperative measurement using the selection guide, which led to oversizing, and 3D CT measurements of the LAA ostium. In contrast to intraoperative measurement, a preoperative surgeon's view 3D VR analysis of the LAA offers a clear view of the LAA base and allows for easy measurement, without the nuisance of a beating heart. As such, surgeon's view 3D VR can potentially provide us with a more realistic estimate of actual LAA base size than intraoperative measurement can. This in turn could prevent rare but serious complications of oversizing and undersizing of the AtriClip; although in our small cohort of 15 patients, we did not encounter any complications related to oversizing the AtriClip. Based on the surgeon's view 3D VR measurements (median 25.7 mm) performed in this study, one could argue that an AtriClip size of 35 mm is always sufficient. However, we believe that in some cases, a 40 mm or 45 mm clip might be a good alternative, since this will most likely facilitate an easier and less forced placement of the AtriClip on the LAA, especially in cases in which the apex of the atrial appendage is larger than the base.

In addition, we experienced other advantages of using 3D VR compared with 2D CT. First, 3D VR allows the user to interactively evaluate the anatomy, since the user is fully submerged into the CT scan. This enables fast recognition of important anatomical structures surrounding the LAA, such as the left main coronary artery, circumflex artery, and pulmonary artery. Also, complex congenital cardiac defects and other anatomical variants can more easily be identified. The 3D VR imaging can also be useful in surgical planning, particularly for determination of thoracoscopic port placement.^9^ Finally, VR offers the ability to simulate procedural steps, which could reduce the learning curve for medical students and residents. Multiple articles have described similar and additional advantages of 3D VR in cardiothoracic residential training, such as the ability to rehearse procedures risk free.^8,12–14^

A limitation of this study is the relatively small sample size. However, our introductory aim with this research does not necessitate a large sample size, and therefore, our findings are still adequately presenting feasibility and applicability. Another limitation of the current study is the use of segmentation files in 3D VR. Segmentation is used to clearly mark the LAA ostium and wall in 3D VR but not in 2D CT. Since segmentation is associated with a loss of precision, a small difference between 3D VR and 2D CT is inevitable. However, this minimal discrepancy, as we have shown in our study, was not significant and probably not clinically relevant.

## Conclusions

3D VR is a feasible method of gaining information on the anatomy and size of the LAA. We hypothesize that the AtriClip is often oversized following intraoperative measurement of the LAA base. Preoperative surgeon's view 3D VR measurement is potentially a more accurate representation of the actual LAA base size than intraoperative measurement. Although our data suggest that for sizing, intracardial 3D VR is not significantly superior to 2D CT, 3D VR could assist in preoperative LAA base sizing to reduce oversizing and undersizing of the AtriClip and related rare complications. In this context, we do believe that there might be an additional value of the surgeon's view 3D VR, because this will facilitate a more detailed preoperative review (compared with 2D CT) of the shape and size of the LAA. In addition, in 3D VR, the anatomy of the LAA is much clearer and more intuitively presented than in 2D CT, which allows for easy recognition of important structures. More importantly, 3D VR could be of enormous value in the education and training of residents. Further research into these domains will be needed, to fully discover and employ the abilities of 3D VR in cardiothoracic surgery.

## Supplemental Material

Visual abstract - Supplemental material for Immersive 3D Virtual Reality–Based Clip Sizing for Thoracoscopic Left Atrial Appendage Closure Click here for additional data file. Supplemental material, sj-pptx-1-inv-10.1177_15569845221114344 for Immersive 3D Virtual Reality–Based Clip Sizing for Thoracoscopic Left Atrial Appendage Closure by Frank van Schaagen, Yvar P. van Steenis, Amir H. Sadeghi, Ad J.J.C. Bogers and Yannick J.H.J. Taverne in Innovations: Technology and Techniques in Cardiothoracic and Vascular Surgery
