# Supplementary material for: Immersive 3D Virtual Reality–Based Clip Sizing for Thoracoscopic Left Atrial Appendage Closure
Source: Innovations (Phila). 2022 Aug 1;17(4):304–9. doi: 10.1177/15569845221114344 (PMC9403374; doi:10.1177/15569845221114344)
Supplement: Visual abstract - Supplemental material for Immersive 3D Virtual Reality–Based Clip Sizing for Thoracoscopic Left Atrial Appendage Closure [file sj-pptx-1-inv-10.1177_15569845221114344.pptx]

## Slide 1
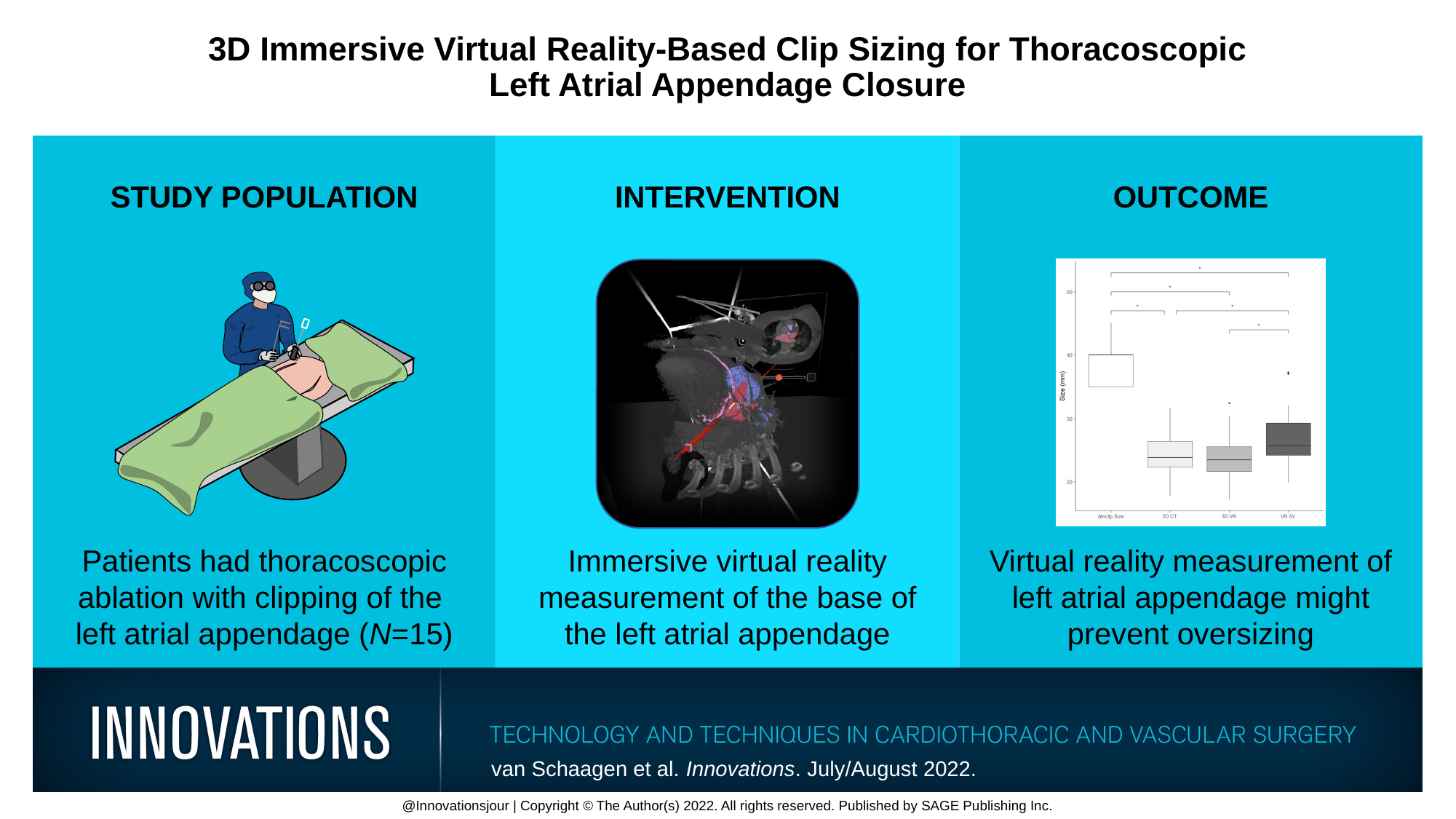

# 3D Immersive Virtual Reality-Based Clip Sizing for Thoracoscopic Left Atrial Appendage Closure
STUDY POPULATION
Patients had thoracoscopic ablation with clipping of the
left atrial appendage (N=15)
INTERVENTION
Immersive virtual reality measurement of the base of
the left atrial appendage
OUTCOME
Virtual reality measurement of left atrial appendage might prevent oversizing
van Schaagen et al. Innovations. July/August 2022.
@Innovationsjour | Copyright © The Author(s) 2022. All rights reserved. Published by SAGE Publishing Inc.
